# To eat, or not to eat: a phantom decoy affects information-gathering behavior by a free-ranging mammalian herbivore

**DOI:** 10.1093/beheco/arad057

**Published:** 2023-07-27

**Authors:** Cristian Gabriel Orlando, Peter B Banks, Tanya Latty, Clare McArthur

**Affiliations:** School of Life and Environmental Sciences, The University of Sydney, Heydon-Laurence Building A08, Science Rd., Camperdown, Sydney, NSW 2050, Australia; School of Life and Environmental Sciences, The University of Sydney, Heydon-Laurence Building A08, Science Rd., Camperdown, Sydney, NSW 2050, Australia; School of Life and Environmental Sciences, The University of Sydney, Heydon-Laurence Building A08, Science Rd., Camperdown, Sydney, NSW 2050, Australia; School of Life and Environmental Sciences, The University of Sydney, Heydon-Laurence Building A08, Science Rd., Camperdown, Sydney, NSW 2050, Australia

**Keywords:** decision-making process, food choice, heuristics, natural background context, swamp wallaby *Wallabia bicolor*

## Abstract

When foraging, making appropriate food choices is crucial to an animal’s fitness. Classic foraging ecology theories assume animals choose food of greatest benefit based on their absolute value across multiple dimensions. Consequently, poorer options are considered irrelevant alternatives that should not influence decision-making among better options. But heuristic studies demonstrate that irrelevant alternatives (termed decoys) can influence the decisions of some animals, indicating they use a relative rather than absolute evaluation system. Our aim was to test whether a decoy influenced the decision-making process—that is, information-gathering and food choice—of a free-ranging mammalian herbivore. We tested swamp wallabies, *Wallabia bicolor,* comparing their behavior toward, and choice of, two available food options over time in the absence or presence of the decoy. We used a phantom decoy—unavailable option—and ran two trials in different locations and seasons. Binary preferences (decoy absent) for the two available food options differed between trials. Irrespective of this difference, across both trials the presence of the decoy resulted in animals more likely to overtly investigate available food options. But, the decoy only shifted food choice, weakly, in one trial. Our results indicate that the decoy influenced the information-gathering behavior during decision-making, providing the first evidence that decoys can affect decision-making process of free-ranging mammalian herbivores in an ecologically realistic context. It is premature to say these findings confirm the use of relative evaluation systems. Whether the foraging outcome is more strongly affected by other decoys, food dimensions, or ecological contexts, is yet to be determined.

## Teaser

Classic foraging models assume that poorer food options are irrelevant when animals make decisions about better food options. However, we found wild foraging wallabies increase information-gathering behaviors by investigating available food options more in the presence of a “phantom decoy”— unavailable option—that was desirable. Our finding—that an irrelevant option affects behaviors during decision-making—brings us closer to understanding cognitive processes behind food selection and may improve predictive capacity of ecological foraging models.

## INTRODUCTION

Foraging is a crucial determinant of an animal’s survival and fitness. It requires significant time investment, and its outcome limits the energy available for other activities. Many factors can affect decisions of foragers, including both intrinsic factors such as hunger level (Talling et al. 2002), sex (Di Stefano et al. 2009) or personality (Mella et al. 2015; Herath et al. 2021), and extrinsic factors such as predation (Palmer et al. 2017) and thermal risks (Trillmich and Trillmich 1986). But, the most obvious factors affecting foraging decisions are associated with foods themselves, such as their abundance, nutritional value, and toxic costs, and the effort required by foragers to handle and process them (e.g., Wiggins et al. 2003; Marsh et al. 2007; Frye et al. 2013).

Classic ecological foraging theories assume foragers make decisions based on absolute values of food options across these multiple dimensions. The assumption of an absolute evaluation system underpins models based on foragers having perfect, prescient knowledge of the environment (Optimal Foraging Theory; Charnov 1976a, 1976b; Pyke et al. 1977). It is also implicit in more recent Bayesian up-dating foraging models (McNamara et al. 2006) in which animals up-date their imperfect knowledge while foraging, using experience (e.g., Ranc et al. 2020), cues (e.g., McArthur et al. 2019) and/or responses to physiological feedback from the foods they consume (e.g., Higginson et al. 2018).

But making decisions based on absolute values may not be possible, or adaptive, if animals have imperfect knowledge and a limited capacity for processing information (Marsh 2002). Free-ranging foragers in natural landscapes usually have the choice of many multidimensional food options, requiring significant amounts of information to process, compare and decide what to eat. Complex decisions and limited information may lead foragers to use simple heuristics (i.e., rules of thumb; Hutchinson and Gigerenzer 2005) to make decisions.

Heuristics can be beneficial by speeding up decision-making and saving energy by requiring less information, hence less cognitive power, without necessarily losing much accuracy (Marsh 2002; Fawcett et al. 2014). Heuristics can also lead to different decision outcomes to those based on absolute values by making decisions based on relative values—termed context-dependent decisions (Morgan et al. 2012; Noguchi and Stewart 2014). The evaluation system foragers use influences the fitness of individuals (Waksberg et al. 2009; Fawcett et al. 2014). Thus, understanding whether animals use relative or absolute evaluation system is important from cognitive, ecological, and evolutionary perspectives.

One form of evidence that animals use heuristics, and a relative evaluation system, is to test their response to an irrelevant alternative when making decisions. An irrelevant alternative—termed a decoy—has an absolute value that is lower than other items in the choice set. Decisions about better options should therefore be unaffected by decoys if these decisions are based on absolute values. In contrast, if a decoy alters the response of decision-makers to better options, this is evidence of a relative evaluation system (Pettibone and Wedell 2007).

Asymmetrically dominated decoys and phantom decoys are two types of decoys often used in studies of heuristics. An asymmetrically dominated decoy (e.g., (Bateson et al. 2003)) is worse in all relevant dimensions (e.g., nectar volume and nectar concentration for pollinators) than one option within a choice set and better than a second option only in some dimensions. A phantom decoy is an option that is unavailable at the time of choice (Pratkanis and Farquhar 1992), and so its actual value is zero. An example is an item advertised to people that is actually out of stock. Effects are generally tested with a highly attractive phantom decoy (e.g., Scarpi 2011; Trueblood and Pettibone 2017; Maia and Volpato 2019), but unattractive ones can also be used (Tan et al. 2015).

Studies with humans (Pettibone and Wedell 2007; Scarpi and Pizzi 2013; Trueblood and Pettibone 2017) and with captive animals—for example, fish (Maia and Volpato 2019), cats (Scarpi 2011), and monkeys (Parrish et al. 2015)—have shown that decoys can change relative preferences of better options, but not always (e.g., Hemingway et al. 2017). However, decisions in natural habitats, including foraging decisions, are much more complex than those in controlled conditions testing captive animals. Natural habitats have a great variety of multidimensional options, the ecological background is dynamic, and from the foraging perspective the nutritional state of animals varies not only among individuals but also over time for any individual. Thus, decision-making by wild animals may differ from those in captive conditions. The few studies on free-ranging animals (e.g., with birds and bees, Shafir et al. 2002; Bateson et al. 2003) have also shown a decoy effect. Studies with a variety of species in natural conditions are needed to gauge any generality in the influence of decoys on foraging by free-ranging animals. If decoys do alter choices among better food options, then incorporating their influence would improve our understanding of foraging, and the predictive capacity of foraging models.

**Figure 1:**
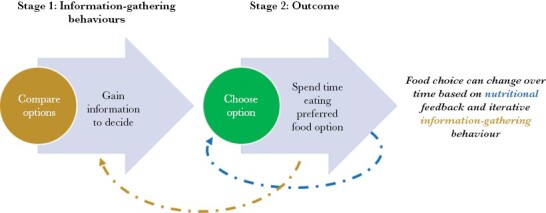
Stages 1 and 2 of the decision-making process during foraging. Decoys may affect one or both stages.

To date, the focus in testing the impact of decoys on foraging animals has been on the decision outcome, that is, food choice. However, the decision-making process is more complicated than just the outcome (Figure 1). Behaviors preceding and affecting choices may also be altered by decoys. For example, studies tracking eye movement to examine behavioral stages in decision-making show that people compare attributes of available options to make decisions, altering which options they look at, how long they look for, and how often they switch, depending on choices on offer (Noguchi and Stewart 2014). Quantifying behaviors, such as how information is being gathered, helps us understand how the decision is being made. To the best of our knowledge, no study has examined these behaviors during decision-making in free-ranging animals.

Here, our aim was to test whether a phantom decoy influenced behavior during the decision-making process, and the food choice outcome, of a free-ranging mammalian herbivore. We tested food choice at three timepoints within a feeding bout. Decoy studies usually consider a decision at a single timepoint, often the first item selected. But for animals such as herbivores, food choice during a feeding bout may change over time, and so may any impact of a decoy. We ran the experiment as two trials, in different locations and seasons. This allowed us to test whether decision-making, food choice, and any effect of the decoy was consistent over space and time irrespective of the background ecological context.

We used a phantom decoy for three reasons. First, they are ecologically relevant as they are present in many forms in nature—for example, the smell of a food item out of reach, a food item that appears rewarding but is empty (e.g., nectarless flowers encountered by foraging bees, Tan et al. 2015), or a food being consumed by a neighbor. Second, any influence they have on decision outcomes is not confounded by them changing the nutritional state of animals because they cannot be consumed. Third, no “random dilution” effect is possible (see Bateson 2002)—as phantom decoys cannot be chosen.

We used free-ranging swamp wallabies (*Wallabia bicolor*), as an example of a generalist herbivore, in their native *Eucalyptus* forest environment. Generalist herbivores frequently encounter and must choose amongst a variety of food types (e.g., plant species) that are in close proximity and that differ in many dimensions, such as nutrition, toxicity, and handling time. They also make thousands of dietary choices daily (Senft et al. 1987), and every time they decide to eat one food item over another, they define their nutritional intake and reduce the time they can spend eating other options. Their foraging decisions also have a substantial impact on ecosystems around the world, particularly with the loss of apex predators (Morgan 2021). Wallabies in Australia, deer in North America, Europe, and Asia, antelope and elephants in Africa define the growth, survival, or death of many plants in their path, shaping plant communities, and indirectly altering other significant components of the ecosystem (Rooney 2001; Guldemond and Van Aarde 2008; Foster et al. 2016; Reed et al. 2022). Yet whether foraging by these herbivores is influenced by decoys is unknown.

## MATERIALS AND METHODS

### Study site and study species

We studied wild swamp wallabies in Ku-ring-gai Chase National Park (Sydney, Australia). Vegetation in this park includes eucalypt woodland, with species such as *Eucalyptus haemastoma* and *Corymbia gummifera*, and open forest with species such as *Eucalyptus paniculata* and *Allocasuarina torulosa* (NSW National Parks and Wildlife Service 2002).

### Selecting and preparing appropriate food options

Decoy experiments typically use two available options and the decoy. The two available options are often defined in two dimensions (e.g., two nutrients) and are chosen to be equally preferred in the binary comparison (decoy absent). For the phantom decoy, a third food option is present but is itself unavailable. We chose to use a commonly used phantom decoy, that is, one that was superior to the available options on at least one dimension (e.g., Trueblood and Pettibone 2017). Any impact of the decoy is tested by comparing the response of the decision-maker to the two available food options between binary (decoy absent) and trinary (decoy present) treatments.

A previous study (Bedoya-Pérez, Issa, et al. 2014) has shown that food preference of free-ranging swamp wallabies can be quantified as a function of two dietary dimensions: nitrogen (N, indicating nutritional level) and 1,8-cineole (a terpene found in *Eucalyptus* leaves indicating toxicity level (Moore et al. 2004; Batish et al. 2008)). Here, we used artificial food to precisely control these beneficial nutritional and costly toxicity levels. To create our food, we used combinations of ground commercial rabbit pellets (either “Nibbler Rabbit & Guinea Pig Lucerne Pellets” 3.07% N, or “Vetafarm Rabbit Origins Rabbit Food” 2.49% N) and oaten hay (either 0.64% N as % of dry matter for > 2 years old hay, or 1.17% N for new hay). We used water (~80 ml per 20 g of dry matter) to mix the ground pellets and hay into a homogeneous paste, then added the required amount of 1,8-cineole (Sigma-Aldrich Pty Ltd, North Ryde, NSW, Australia) to each food option. To conserve food properties and limit cineole and water evaporation, the food was prepared on the morning of the day it was used. From an ecological perspective, all the diets we tested in the pilot and main study were chemically realistic, with nitrogen and cineole concentrations within the natural range in plants typically consumed by wallabies. While we describe the foods on these two dimensions, they varied in other dimensions, such as fiber and micronutrients as well.

**Figure 2:**
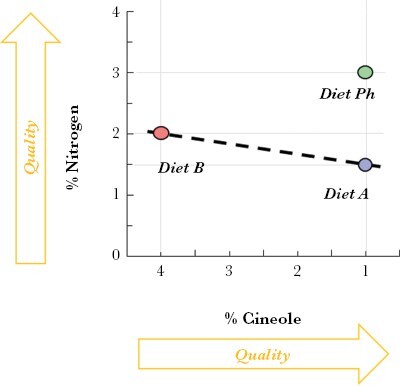
Plot showing our food options based on two important dietary dimensions (nitrogen and cineole concentration) that affect food preference by swamp wallabies. The X-axis is inverted as an increase in cineole is negatively correlated with food quality. The dashed line indicates combinations of cineole and nitrogen that should produce equally preferred food based on results from (Bedoya-Pérez, Issa, et al. 2014).

To select appropriate food options for the experiment, we based our choices on (1) preference results for different combinations of nitrogen-cineole in food tested in pilot studies; and (2) previous results on food preference of free-ranging swamp wallabies at the same study site (Bedoya-Pérez, Issa, et al. 2014). For the two equally preferred available food options (Figure 2), we selected a low-nitrogen low-cineole food option (*food A:* 1.5% N, 1% cineole) and high-nitrogen high-cineole food option (*food B:* 2% N, 4% cineole). Our best option for the decoy was a high-nitrogen low-cineole food (*food Ph:* 3% N, 1% cineole). We added cineole in low concentration, as a non-zero amount on the cineole axis that did not reduce food intake (Bedoya-Pérez, Isler, et al. 2014) hence representing minimal cost. The decoy *food Ph* was nutritionally superior to both *food A* and *food B* on the nitrogen axis, particularly important for herbivores since they typically consume a low nitrogen food, and it was superior to *food B* but not *food A* on the toxic (cineole) axis (Figure 2). In the pilot study, *food Ph* was preferred over *food A* but was similarly preferred to *food B*.

### Presentation and comparison of treatments

**Figure 3:**
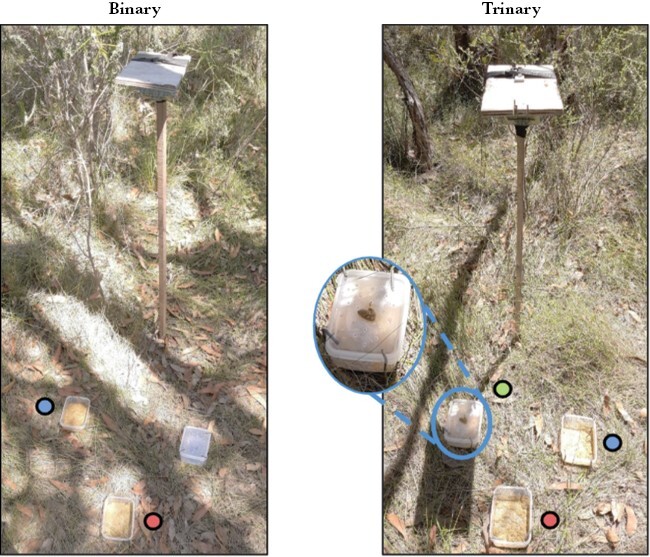
Set up of the two treatments. On the left, the binary treatment including *diet A* (in blue), *diet B* (in red) and an empty container. On the right, the trinary treatment including *diet A* (in blue), *diet B* (in red), and *diet Ph* (the phantom decoy, in green). *Diet Ph* is covered by a perforated lid, with only half a teaspoon of food on top.

We compared two treatments (Figure 3): *binary* with two available food options (decoy absent) versus *trinary* with two available food options as for binary plus the phantom decoy (present but unavailable). For the *binary treatment:* the two available food options (*food A* and *food B*, 20 g dry weight of each) were presented along with an empty plastic container with a transparent perforated lid. We used 20 g (<0.2% of animal weight) for each food option, so its consumption during a visit would have a negligible impact on an animal’s nutritional state and future preference. Options within a treatment were arranged in a triangle, ~25 cm apart, close enough so animals could smell or easily reach all options from any position. In the *trinary treatment* the phantom decoy (*food Ph,* 20 g) was presented in the container with the transparent perforated lid so animals could see and smell the food but not access it. An important consideration here is that swamp wallabies use food odor cues to find and select preferred foods (Stutz et al. 2016; Finnerty et al. 2017; Orlando et al. 2020). We know that foods similar to those we used here emit odors, and that another herbivore (common brushtail possum *Trichosurus vulpecula*) can use the odors as a cue for differentiating nutritional content of the foods in the absence of 1,8-cineole (Mella et al. 2018). However, the strong odor of 1,8-cineole may hinder the olfactory capacity of wallabies (Bedoya-Pérez, Isler, et al. 2014) to detect differences in nutritional content of the foods. We therefore placed half a teaspoon of our phantom decoy on top of the lid, with the remainder unavailable inside. This amount was negligible compared to the other available food options, yet it meant an animal could sample the decoy and have the chance to associate its quality with the unavailable food inside the container. This set-up is effectively an unrecognized (or unknown) phantom decoy: the consumer does not know the option is unavailable until after trying to get it (Pratkanis and Farquhar 1992; Scarpi 2011).

### Experimental design

We ran the experiment twice in the same way; *trial 1* in February (summer) and *trial 2* in July (winter) in different locations within the study site (~150–750 m distance between the two). We did this for logistical reasons, but it also meant we could test whether food preferences and any effect of the decoy were consistent irrespective of time and place. Wallabies alter their diet depending on the frequencies of food types in different habitats (Di Stefano and Newell 2008) and seasons (Hollis et al. 1986), and nutritional requirements and hunger of animals can vary over time and space. Such changes in ecological context could alter the perceived or absolute value of food options and, conceivably, the effect of decoys.

We used a randomized block design for each trial, with six blocks for *trial 1* and nine blocks for *trial 2*. Blocks were at least 200 m apart to increase the chances of different animals visiting different blocks based on their home range and overlap (Ben-Ami 2005). Within each block, we allocated two plots, 15 m apart, so plots within a block were surrounded by similar vegetation and conditions. We randomly allocated the *binary* treatment to one of the plots within a block and the *trinary* treatment to the other. When considering all first visits of each night in *trial 1* and *trial 2*, the total number of times the *binary* or *trinary* treatment was visited first in the blocks was similar: 51% versus 49%, respectively. Thus, even if the same wallaby moved from one plot to the next within a block, the order of visits was balanced between treatments.

Trial 1 ran over 3 days and trial 2 ran over 3–4 days depending on the block (4 days if rain damaged food before a visit in one of the previous days). Each day, we switched the *binary* and *trinary* plots within each block and rotated the position of the food options clockwise within plots (the first food option position was randomly selected at each plot). These changes were made to prevent any effect of food position (e.g., if the choice was affected by which option the wallaby encountered first; Evans et al. 2021), or learning, on an animal’s decisions, for example, if the animal always approached the plot from the same side. All food options were replaced each day, so containers were full for the first visitor of that day and cleaned of any odor of other animals to prevent any conspecific odor cues affecting an animal’s decision.

### Quantifying behavior during decision-making and food choice outcomes

We placed a motion-triggered infrared camera (ScoutGuard SG2060-K) to record visits of wallabies to the plots, their behaviors, and feeding. Cameras were positioned next to each feeding station (Figure 3) at a height of ~1.25 m, and with a slight inclination, to include the three food options and close surroundings in the camera’s field of view. The cameras were set up to record 1-min videos, with no gap between videos, for 24 h. For each plot, we only used the first visit of each night in which an animal ate any of the food options, as the results of following visits may be affected by the amount of food remaining and the presence of odor cues from conspecifics. We included all *trinary* first visits, irrespective of whether the animal had or had not overtly investigated the phantom decoy before eating from the other two food options because wallabies can detect odor cues from afar (Stutz et al. 2017; Orlando et al. 2020) and, thus, the odor from the phantom decoy could still be processed and affect decision-making.

For each visit, we only analyzed the first 5 min, as in the pilot study, this was the average time it took animals to deplete one of the food options if it focused on eating that food option alone. During these 5 min, we first quantified one behavior, termed *Compare both*, as a form of information-gathering comparative assessment during decision-making (Figure 1, stage 1). *Compare both* quantified whether (binary data, yes/no) the animal overtly smelled and/or ate from *both* food options (A and B) at any time during the 5 min of the visit. We considered the animal was smelling if it clearly pointed its snout at one of the containers or moved its head as if sampling the air. We considered the animal was eating when its snout was inside one of the plastic containers in contact with the food. This *Compare both* variable was chosen to quantify information-gathering behavior similar to eye movement tracking with people (Noguchi and Stewart 2014). It was more appropriate for wallabies because they use odor, rather than vision, as their main foraging cue for assessing food quality before choosing (i.e., before tasting/consuming; Finnerty et al. 2017; Stutz et al. 2017).

Next, we quantified the choice outcome (Figure 1, stage 2) using preference at three timepoints during a visit: instantaneous, short-term, and longer-term. All preferences were represented as binary data with reference to *food A*. *Instantaneous preference* was the first food eaten (A or B; as A yes/no). *Short-term preferenc*e was the food option (A or B) eaten during the first 20 s of eating (excluding time dedicated to other behaviors). In several cases, both food options (A and B) were eaten. If the proportion from one of the options was > 0.6 of the total time spent eating, we defined it as preferred (similar to criteria used by Scarpi 2011). In two cases (for *trinary* in *trial 1*), the proportion was 0.5 ± 0.1. These were considered equally preferred, and we excluded them from the analysis. *Longer-term preference* was the food option eaten most during the first 5 min of the visit (including time dedicated to other behaviors). If more than one food option was consumed during this time, we used the same rule as for *short-term preferenc*e to define preference as a binary response. In one visit (for *trinary* treatment in trial *1*), both food options were equally preferred and this was excluded. For those visits (*n* = 13 out of 79), where less than 40 s (double the time used for *short-term preference*) of an animal eating was recorded on camera out of the 5-min visit (e.g., because the wallaby did not move enough to re-trigger the camera), we were able to use videos following the first 5 min to corroborate the longer-term preference. That is, if we could clearly determine that the animal was still in the same position as it was in the last videos within the 5-min visit, and eating from the same container, the longer-term preference was confirmed. If a confirmation was not possible (e.g.,because no more videos were available or because next available video showed both food were depleted), the video was excluded from the analysis.

### Statistical analysis

We analyzed the data using generalized linear mixed models with a binomial distribution and logit link function (all response variables were binary) using the lme4 package (Bates et al. 2015) in RStudio (RStudioTeam 2021). Output of the final models were plotted using ggplot2 (Wickham 2016) and visreg (Breheny and Burchett 2017) packages in RStudio.

We started with full models of fixed and random explanatory variables. The fixed factors were treatment (*binary* or *trinary*), trial (*1* or *2*), plot visit order (whether a plot within a block was visited first or second in the same night, not necessarily by the same animal), investigate Ph/empty 1st (whether the phantom decoy or empty container was overtly investigated before eating one of the available food options), total time eating (*foods A* and *B* during the 5-min visit), and day (1–4). Random factors were block and plot (nested within block).

First, we tested whether the two available foods (A and B) were equally preferred in the binary treatment across *trial 1* and *trial 2*, as is typical in decoy trials. We tested this with longer-term preference data (the timeframe showing the greatest change between trials) and the binary treatment only.

The full model was:

*Model 1: food A* preferred (1,0) = trial + plot visit order + investigate Ph/empty 1st + total time eating + day + (1|block) + (1|block:plot)

Next, using the binary and trinary treatments, we tested the impact of the decoy on

(1) Model 2: the behavior *Compare Both* (Figure 1 stage 1), and
(2) Model 3: food choice outcome (preference relative to food A, Figure 1 stage 2) at each of the three timepoints (*instantaneous*, *short*- and *longer-term*).

The full models were:

*Model 2: Compare Both* = treatment × trial + plot visit order + investigate Ph/empty 1st + total time eating + day + (1|block) + (1|block:plot)

Model 3 [separately for trials 1 and 2]: *food A* preferred (1,0) = treatment × investigate Ph/empty 1st + plot visit order + total time eating + day + (1|block) + (1|block:plot)

For Model 2, we kept the *Compare both* data pooled across trials and tested the interaction between treatment and trial. We did so because this behavior, as stage 1 of the decision-making process, is used by the subject in determining the outcome (stage 2), irrespective of what that outcome is. We analyzed Model 3 separately for trials 1 and 2, because any impact of the decoy on food choice may be differentially affected by the difference in the binary preference between these trials (from Model 1, see results). For Model 3, we included the interaction between “treatment” and “investigate Ph/empty 1st” in the full model because the potential information wallabies could obtain from overtly investigating the empty container (“binary” treatment) versus the Phantom decoy container (“trinary” treatment) differed.

Using the Akaike information criterion, we confirmed that neither of the random factors improved the models (covariance at the block or plot within the block level was trivial). We then reduced the full models to the most parsimonious fixed effect model for each response variable (see models in the results section of the Supplementary Material).

## RESULTS

For *trial 1*, there were 14 visits by wallabies to the *binary* treatment and 16 visits to the *trinary* treatment from which we could retrieve data for our variables. For *trial 2*, there were 22 visits for *binary* and 27 visits for *trinary* treatment. From all these visits, our total sample sizes for each variable was *n* = 77 for *compare both* behavior, *n* = 75 for instantaneous preference (27 for *trial 1* and 48 for *trial 2*), *n* = 70 for short-term preference (23 for *trial 1* and 47 for *trial 2*) and *n* = 75 for longer-term preference (27 for *trial 1* and 48 for *trial 2*).

### Binary food preference across trials 1 and 2

**Figure 4:**
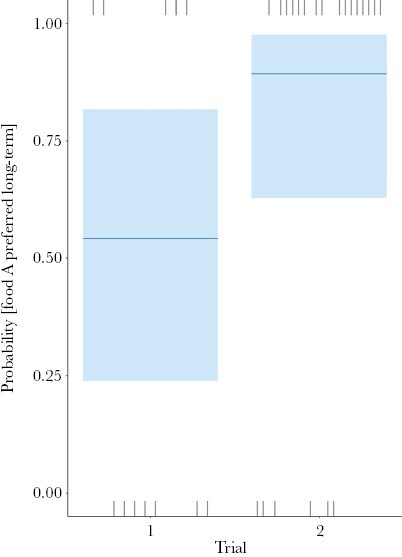
Probability that wallabies preferred food A over food B in the longer-term, for each trial in the binary treatment only. Plot shows confidence bands, median, and observations (rugs).

In the *binary* treatment only, the longer-term preference for *food A* differed significantly between trials (LR χ^2^_(1, *N* = 36)_ = 5.08, *P* = 0.024). In trial 1, the probability of A being preferred over B was similar to 0.5, but in trial 2, the probability of A being preferred over B was much higher (median near 0.9; Figure 4). *Plot visit order* also had a significant effect on longer-term preference (LR χ^2^_(1, *N* = 36)_ = 4.859, *P* = 0.028). A modified model including the interaction between *plot visit order* and *trial*, showed this interaction was not significant (LR χ^2^_(1, *N* = 36)_ = 1.829, *P* = 0.1768).

### Binary versus trinary treatments: Information-gathering behavior (stage 1)

**Figure 5:**
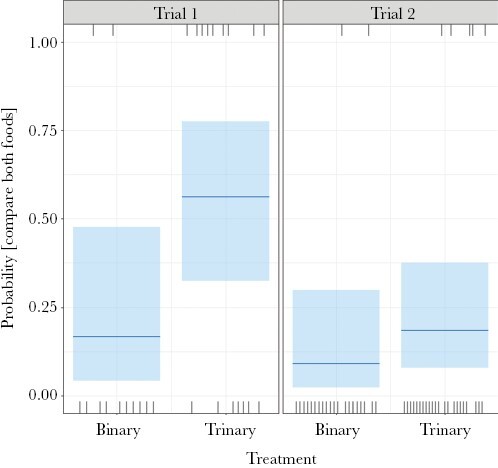
Probability that wallabies overtly compared both available foods in each treatment for trial 1 and 2. Plot shows median with confidence bands, and each observation (rugs).

The probability that wallabies overtly compared both available foods (*Compare both* variable) was significantly higher when the phantom decoy was present (χ^2^_(1, *N* = 77)_ = 5.038, *P* = 0.025, Figure 5). *Trial* also had a significant effect on *Compare both* behavior (χ^2^_(1, *N* = 77)_ = 6.223, *P* = 0.013), but the interaction with treatment was not significant (see results in the Supplementary Material). The probability of comparing both available foods in trial 1 was higher than in trial 2.

### Binary versus trinary treatments: food choice (stage 2)

**Figure 6:**
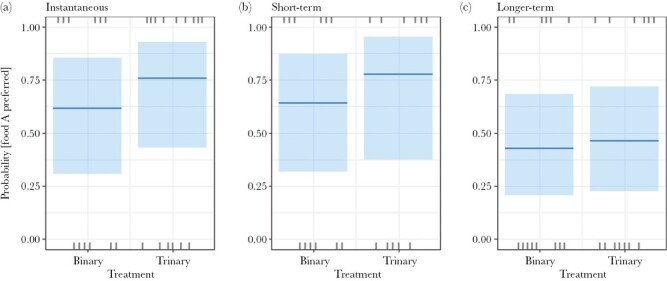
Probability that wallabies preferred food A over food B for the (a) instantaneous, (b) short-term and (c) longer-term preferences in trial 1. Plot shows median with confidence bands, and each observation (rugs).

In *trial 1*, there was no significant effect of treatment on the instantaneous (χ^2^_(1, *N* =27)_ = 0.638, *P* = 0.425), short-term (χ^2^_(1, *N* = 23)_ = 0.483, *P* = 0.487), or longer-term (χ^2^_(1, *N* = 27)_ = 0.03, *P* = 0.863) preference for *food A* (compared with *food B*; Figure 6). *Plot visit order* showed a significant effect on short-term preference (see results in the Supplementary Material)).

**Figure 7:**
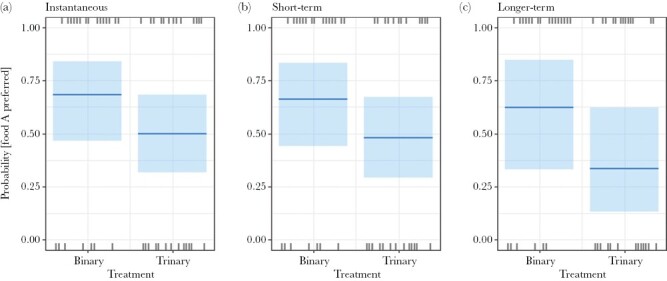
Probability of preferring food A for the (a) instantaneous, (b) short-term and (c) longer-term preferences in *trial 2*. Plot shows median with confidence bands, and each observation (rugs).

For *trial 2*, there was no statistically significant effect of treatment on the instantaneous (χ^2^_(1, *N* = 48)_ = 1.567, *P* = 0.211), short-term (χ^2^_(1, *N* = 47)_ = 1.39, *P* = 0.238), or longer-term (χ^2^_(1, *N* = 48)_ = 3.29, *P* = 0.07) preference for *food A* compared with *food B* (Figure 7). But for longer-term preference, median probability that food A was preferred dropped from ~0.6 in the binary treatment to ~0.3 in the trinary treatment (Figure 7c).

## DISCUSSION

In this study, we quantified the impact of a phantom decoy on information-gathering behavior and food choice outcomes at three timepoints by free-ranging swamp wallabies. We ran the experiment twice, in two trials at different times and in a different place. Despite offering the same foods, the preferences in the binary treatment at the population level differed between trials. In trial 1, foods A and B were similarly preferred by wallabies; but in trial 2, wallabies strongly preferred food A.

Our results show that the decoy had a significant impact on information-gathering behavior (stage 1 of decision-making process) across trials, increasing the probability that wallabies overtly compared both available foods. When analyzing the decision-making outcome (stage 2 of the decision-making process), in trial 1, the decoy had no impact on food choice at any timepoint. But in trial 2, the decoy had a weak effect (although not statistically significant) on longer-term preference, with the strong preference for food (A) in the binary treatment (decoy absent) reduced in the trinary treatment (decoy present). While we used an attractive phantom decoy, typical of many studies on decoys, effects of an unattractive phantom decoy are also worth exploring in future studies.

### Binary preferences of free-ranging animals differ in space and time

Our results show that food preference in swamp wallabies is affected by the background context, as the preference for *food A* and *B* in the binary treatment differed between trials. From an ecological perspective, this difference is not surprising and could have arisen for several reasons. For example, differences in the availability of resources (i.e., different background vegetation) can affect the demand for nutrients (Di Stefano and Newell 2008); differences in the nutritional state of the different animals can affect their nitrogen demand and energy available for detoxification (Parikh et al. 2017); or there may be different nutritional needs of the animals associated with, for example, sex (Kie and Bowyer 1999; Di Stefano et al. 2009) or breeding season.

A change in food preference between trials also demonstrates the added complexity of testing impacts of decoys in the real world with free-ranging animals compared to the typical controlled environment of captive animal studies. Whether the relative preference for the available options alters any effect of decoys is unknown, because decoy studies generally choose two foods of equal preference. But since many food options in choice sets of foragers are not equally preferred, the ecological relevance of decoy theory would increase by incorporating the impact of non-equal “starting” binary preferences into its framework. Future decoy studies could test the consequences of using non-equally preferred available options on the effects of decoys on decision-making. We inadvertently started to test this in trial 2, where food A was strongly preferred over food B in the binary treatment by the wallaby population as a whole. A decoy may more readily affect food choice, and/or its effect may be easier to detect statistically, when most animals prefer the same food in the binary treatment, provided the decoy acts to shift a population’s preference away from this food. Such a conclusion is consistent with the difference in longer-term preference results between our trials 1 and 2, with and without the decoy (Figures 6c and 7c).

### Impact of the decoy on stage 1 (information-gathering behavior) of decision-making

We found that the probability of wallabies comparing both foods A and B, that is, *Compare both*, during the decision-making process increased significantly when the decoy was present, and was greater in trial 1 than trial 2 (Figure 5). In trial 1, foods A and B were similarly preferred in the binary treatment, while A was much more preferred than B in trial 2 (Figure 4). *Compare both* quantified behavior associated with investigating and assessing the foods, that is, with information-gathering, similar to eye movement patterns in visual species such as humans (Noguchi and Stewart 2014). The increased probability of comparing both available foods in the presence of the decoy suggests that the decoy increased the need for collecting more information during the decision-making process, possibly increasing the cognitive load. The result also suggests that this need for information increased more when the available food options were similarly preferred (trial 1) than when one option was strongly preferred (trial 2). Just as the current decoy theory has yet to incorporate the impact of non-equal preference on the food choice outcome, its impact on cognitive costs and the information-gathering stage of decision-making needs attention. Given many choices in the real world involve options that are not equally preferred, our understanding of the impact of decoys would benefit from doing so.

### Impact of the decoy on stage 2 (food choice outcome) of decision-making

In trial 1, we found no effect of the decoy on food choice at any of the three timepoints when we measured that choice (instantaneous, short-, and longer-term; Figure 6). These results show that, despite the greater probability of overtly gathering information to make the decision (shown in stage 1), the outcome (stage 2) remained unaltered by the decoy.

However, in trial 2, we found evidence for at least a weak effect of the decoy on food choice (Figure 7). The direction of shift was the same for each of the three timepoints (Figure 7a–c) with a diminishing probability that food A was preferred over B in the presence of the decoy. We suspect a Type II statistical error in the longer-term preference results (*P* = 0.07), but a larger sample size in future is needed to confirm this impact.

## CONCLUSION

Our results show an influence of the phantom decoy on the decision-making process by swamp wallabies. This influence was manifest mainly and most strongly during the information-gathering stage 1 rather than the choice outcome stage 2. An increase in the probability of exploring options when the decoy was present is consistent with wallabies using a relative evaluation system to make decisions. If preferences were based on absolute values, the addition of an irrelevant option should not have affected this behavior. Alternatively, the odor of the decoy may have created uncertainty about the value of the two available food options by interfering with the odor information they emit. Such a reduction in “concreteness” of options can reduce the outcome effects of decoys (Spektor et al. 2021). While we cannot discount this second possibility, we think it is unlikely for several reasons. Swamp wallabies were clearly able to demonstrate a preference between two options in the binary treatment (Figure 4, trial 2). They can also detect and use odors at fine spatial scales to find both novel food (Orlando et al. 2020) and preferred plants (Stutz et al. 2016; Finnerty et al. 2017) in the same complex vegetation and olfactory backdrop as we used here. Finally, another olfactory-oriented mammalian herbivore, the African elephant *Loxodonta africana*, successfully detects and distinguishes the odor of a preferred plant species even when it is offered amongst an abundance of other plant species in the same small container (McArthur et al. 2019).

It is too early to say exactly whether and how irrelevant alternatives should be incorporated into classic foraging models in ecology because our current understanding of how, why, and when decoys have any particular effect is insufficient. The next step is to generate evidence with more empirical studies, varying ecological context, foods, and decoys with different dimensions and relative preferences, to test and understand the boundary conditions when the effects of decoys are changed.

Recent discussions highlight the fragility of the impact of decoys, with evidence that different conditions (context) result in decoys nudging decision outcomes in one direction or in the reverse, or have no impact at all (Spektor et al. 2021). Our results across two trials in one study system demonstrate how labile these impacts are. This is especially the case with free-ranging animals where the ecological context varies, where preferences for the same foods are not always equal nor the same over time and space, and where the different stages in decision-making may be more or less affected by decoys. Clearly, there is a gap in our understanding that can only be filled with future experiments—and theory—exploring the consequences of these diverse but realistic conditions.

## Supplementary Material

arad057_suppl_Supplementary_Material

## Data Availability

Analyses reported in this article can be reproduced using the data provided by Orlando (2023).
