# Supplementary material for: To eat, or not to eat: a phantom decoy affects information-gathering behavior by a free-ranging mammalian herbivore
Source: Behav Ecol. 2023 Jul 27;34(5):759–68. doi: 10.1093/beheco/arad057 (PMC10516680; doi:10.1093/beheco/arad057)
Supplement: arad057_suppl_Supplementary_Material [file arad057_suppl_supplementary_material.docx]

**SUPPORTING INFORMATION**

**Results**

Statistical output table of results for the final reduced generalized linear models for each response variable:

**Binary food preference across trials**

*Model 1: food A* preferred (1,0) = trial + plot visit order + total time eating

|  | LR Chisq | Df | Pr(>Chisq) |
| --- | --- | --- | --- |
| trial | 5.0797 | 1 | 0.02421 * |
| plot visit order | 4.8589 | 1 | 0.02750 * |
| total time eating | 1.2577 | 1 | 0.26208 |

**Information-gathering behaviour across trials**

*Model 2:* *Compare Both* = treatment*trial

|  | LR Chisq | Df | Pr(>Chisq) |
| --- | --- | --- | --- |
| treatment | 5.0378 | 1 | 0.02480 * |
| trial | 6.2227 | 1 | 0.01261 * |
| treatment*trial | 0.6516 | 1 | 0.41954 |

***Trial 1 – Food choice outcome***

Model 3 [*Instantaneous preference*]: *food A* preferred (1,0) = treatment + plot visit order

|  | LR Chisq | Df | Pr(>Chisq) |
| --- | --- | --- | --- |
| treatment | 0.6375 | 1 | 0.42460 |
| plot visit order | 3.1681 | 1 | 0.07509 |

Model 3 [*Short-term preference*]: *food A* preferred (1,0) = treatment + plot visit order

|  | LR Chisq | Df | Pr(>Chisq) |
| --- | --- | --- | --- |
| treatment | 0.4825 | 1 | 0.48728 |
| plot visit order | 4.0107 | 1 | 0.04521 * |

Model 3 [*Long-term preference*]: *food A* preferred (1,0) = treatment

|  | LR Chisq | Df | Pr(>Chisq) |
| --- | --- | --- | --- |
| treatment | 0.029671 | 1 | 0.8632 |

***Trial 2 – Food choice outcome***

Model 3 [*Instantaneous preference*]: *food A* preferred (1,0) = treatment

|  | LR Chisq | Df | Pr(>Chisq) |
| --- | --- | --- | --- |
| treatment | 1.6373 | 1 | 0.2007 |

Model 3 [*Short-term preference*]: *food A* preferred (1,0) = treatment + total time eating

|  | LR Chisq | Df | Pr(>Chisq) |
| --- | --- | --- | --- |
| treatment | 1.5189 | 1 | 0.2178 |
| total time eating | 2.2488 | 1 | 0.1337 |

Model 3 [*Long-term preference*]: *food A* preferred (1,0) = treatment + day

|  | LR Chisq | Df | Pr(>Chisq) |
| --- | --- | --- | --- |
| treatment | 3.2897 | 1 | 0.06971 |
| day | 6.3116 | 1 | 0.09740 |
